# Pharmacogenomics of gemcitabine: can genetic studies lead to tailor-made therapy?

**DOI:** 10.1038/sj.bjc.6603860

**Published:** 2007-06-26

**Authors:** H Ueno, K Kiyosawa, N Kaniwa

**Affiliations:** 1 Hepatobiliary and Pancreatic Oncology Division, National Cancer Center Hospital, 5-1-1 Tsukiji, Chuo-ku, Tokyo 104-0045, Japan; 2 Oncology Business Unit, Eli Lilly Japan KK, 1-1-1, Shin Aoyama Bldg. West 22F, 1-1-1, Minami-Aoyama, Minato-ku, Tokyo 107-0062, Japan; 3 Division of Medicinal Safety Science, National Institute of Health Sciences, 1-18-1 Kamiyoga, Setagaya-ku, Tokyo 158-8501, Japan

**Keywords:** gemcitabine, pharmacogenomics, polymorphisms, gene expression, tailor-made therapy

## Abstract

Gemcitabine is a deoxycytidine analogue that has a broad spectrum of antitumour activity in many solid tumours including pancreatic cancer. We have recently carried out a pharmacogenomic study in cancer patients treated with gemcitabine, and found that one genetic polymorphism of an enzyme involved in gemcitabine metabolism can cause interindividual variations in the pharmacokinetics and toxicity of this agent. In this paper, we review recent genetic studies of gemcitabine, and discuss the possibility of individualised cancer chemotherapy based on a pharmacogenomic approach.

With progress in the development of anticancer agents, many cancer patients now benefit from chemotherapy. Before treatment, however, it is difficult to predict whether the selected chemotherapy will be really effective and tolerable to the patient. Therefore, considerable effort has been made to obtain information that could be used to devise tailor-made therapy. Recent progress in molecular biology has revealed that genetic factors can at least partly explain interindividual variations in the efficacy and toxicity of anticancer agents. We have recently carried out a prospective pharmacogenomic study in cancer patients treated with gemcitabine (2′,2′-difluorodeoxycytidine, dFdC), and found that one of the single-nucleotide polymorphisms (SNPs) in the cytidine deaminase gene influences the pharmacokinetics and toxicities of this agent (Sugiyama *et al*, 2007). Gemcitabine is a deoxycytidine analogue that demonstrates broad anticancer activity in various solid tumours, including pancreatic cancer and non-small-cell lung cancer (NSCLC). Because of the widespread use of gemcitabine, a better understanding of the mechanisms determining its activation, and development of resistance against it has been needed, and this has prompted active genetic studies in relation to this agent. In this review, therefore, we focus on genetic studies of gemcitabine that have yielded data potentially useful for the establishment of individualised cancer chemotherapy.

## GEMCITABINE METABOLISM AND MECHANISM OF ACTION

**Figure 1:**
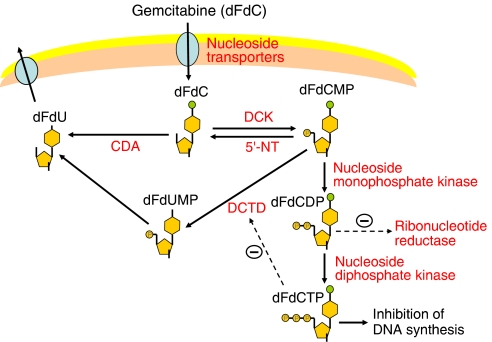
Cellular metabolism and mechanism of gemcitabine. For explanation of symbols and metabolic routes, see text.

Like cytarabine, another widely used nucleoside analogue, gemcitabine is a prodrug that requires cellular uptake and intracellular phosphorylation in order to exert its action (Figure 1) (Fukunaga *et al*, 2004; Mini *et al*, 2006). Once administered, gemcitabine is transported into cells by nucleoside transporters. Gemcitabine is then phosphorylated into gemcitabine monophosphate (dFdCMP) by deoxycytidine kinase (DCK), and dFdCMP is subsequently phosphorylated to gemcitabine diphosphate (dFdCDP) and gemcitabine triphosphate (dFdCTP) by nucleoside monophosphate (UMP/CMP) and diphosphate kinase. Gemcitabine exerts its cytotoxic effect mainly through inhibition of DNA synthesis by being incorporated into the DNA strand as the active dFdCTP. It is known that gemcitabine has a unique mechanism of action known as ‘self-potentiation’ (Heinemann *et al*, 1992). For example, dFdCDP potently inhibits ribonucleotide reductase, resulting in a decrease of competing deoxyribonucleotide pools necessary for DNA synthesis. Again, dFdCTP suppresses inactivation of dFdCMP by inhibiting deoxycytidine monophosphate deaminase (DCTD). On the other hand, more than 90% of administered gemcitabine is converted, and thus inactivated, by cytidine deaminase (CDA) into 2′-deoxy-2′,2′-difluorouridine (dFdU). Phosphorylated metabolites of gemcitabine are reduced by cellular 5′-nucleotidase (5′-NT), and dFdCMP is also converted, and inactivated, by DCTD into 2′-deoxy-2′,2′-difluorouridine monophosphate (dFdUMP).

This paper discusses these various metabolic pathways related to gemcitabine cellular pharmacology and DNA repair. In Table 1, we summarise the genetic polymorphisms related to gemcitabine pathways, their allele frequencies in different ethnic groups, and the resulting functional changes. In this paper, A of the translation initiation codon ATG is numbered 1 and the first methionine of a protein is numbered 1.

## NUCLEOSIDE TRANSPORTERS

Gemcitabine is transported into cells by five nucleoside transporters, two equilibrative nucleoside transporters (ENTs; ENT1 (SLC29A1) and ENT2 (SLC29A2)) and three concentrative nucleoside transporters (CNTs; CNT1 (SLC28A1), CNT2 (SLC28A2), and CNT3 (SLC28A3)) (Mini *et al*, 2006). Kinetic studies of human cell lines have shown that gemcitabine intracellular uptake is mediated mainly by ENT1 and, to a lesser extent, by CNT1 and CNT3.

The reported allele frequencies of nucleoside transporter gene variants are generally low except *ENT1* −706 G>C in Caucasians and *ENT1* −1050 G>A in Africans, as shown in Table 1 (Osato *et al*, 2003; Damaraju *et al*, 2005; Kim *et al*, 2006; Myers *et al*, 2006). To date, it is unclear whether these genetic variants of nucleoside transporter genes including *ENT1* contribute to interindividual differences in response to gemcitabine. The functional analyses of the two nonsynonymous SNPs of *ENT1* (*SLC29A1* 647T>C and 1171G>A) and the three nonsynonymous SNPs of *CNT3* (*SLC28A3* 14G>A, 391 C>T, and 1538A>T) failed to demonstrate functional diversity (Osato *et al*, 2003; Damaraju *et al*, 2005). On the other hand, a recent study found that individuals with CGG/CGC haplotypes based on the three SNPs in the promoter region of *ENT1* (*SLC29A1* −1345C>G, −1050 G>A, −706G>C) showed 1.37-fold higher median expression of the ENT1 transcript than those with the common CGG/CGG haplotypes, suggesting that *ENT1* promoter region variants may influence gene expression and alter gemcitabine chemosensitivity (Myers *et al*, 2006).

As to expression, several studies have suggested that ENT1 expression of mRNA/proteins in tumour tissues may be a good predictive marker of outcome in cancer patients receiving gemcitabine. Spratlin *et al* (2004) performed an immunohistochemical study on paraffin-embedded tumour tissues from 21 patients with pancreatic cancer and reported that overall survival was significantly longer in those expressing detectable amounts of ENT1 in tumour blocks than in those with low or absent ENT1 following gemcitabine treatment (median, 13 months *vs* 4 months; *P*=0.01). Polymerase chain reaction analysis of 81 patients with pancreatic cancer also showed that those with high ENT1 mRNA expression in the tumour specimens had significantly longer survival after gemcitabine therapy than patients with low ENT1 levels (median, 25.7 *vs* 8.5 months; *P*<0.001) (Giovannetti *et al*, 2006). Similar results were obtained in a study of 12 bladder cancer patients treated with gemcitabine, which demonstrated that the mean level of ENT1 mRNA in tumour specimens was significantly higher in patients achieving a complete pathological response than in those with stable disease (1.166 *vs* 1.021; *P*=0.040) (Mey *et al*, 2006). These results suggest that tumour-specific expression of ENT1 may be a promising predictive biomarker of outcome after gemcitabine treatment, although formal validation in prospective studies is needed.

## CYTIDINE DEAMINASE

Cytidine deaminase is involved in the salvaging of pyrimidines, and plays a key role in detoxifying gemcitabine. Therefore, patients with impaired CDA activity might develop strong toxicities after administration of gemcitabine, while CDA overexpression in tumour tissues might reduce the antitumour efficacy of this drug. An *in vitro* study has demonstrated resistance to gemcitabine in cells overexpressing CDA (Neff and Blau, 1996).

So far, two nonsynonymous SNPs, 79A>C (Lys27Gln) and 208G>A (Ala70Thr), have been identified in the coding region of the human CDA gene (Yue *et al*, 2003; Fukunaga *et al*, 2004; Gilbert *et al*, 2006; Sugiyama *et al*, 2007). Ethnic or racial differences in the allele frequencies of these SNPs have been reported, as shown in Table 1. Remarkable reduction in activity of 70Thr CDA was reported *in vitro* (Yue *et al*, 2003) and *in vivo* (Sugiyama *et al*, 2007), while only marginal reduction in activity of 27Gln CDA was observed *in vitro* (Yue *et al*, 2003; Gilbert *et al*, 2006). On the other hand, Fitzgerald *et al* (2006) investigated SNPs in the promoter region of CDA *in vitro* and *in vivo*, and found that some promoter *CDA* haplotypes might affect CDA activity.

**Figure 2:**
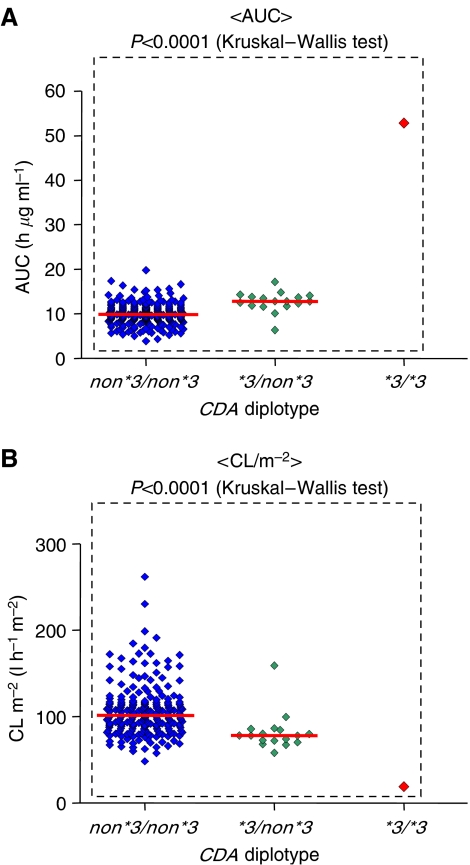
Effects of CDA ^*^3 on the pharmacokinetic parameters of gemcitabine. (**A**) Area under the curve (AUC) and (**B**) clearance (CL m^−2^). Each point corresponds to an individual patient. The bars denote the median values.

With regard to the correlation between *CDA* SNPs and clinical outcome, we have recently carried out a prospective pharmacogenomic study in cancer patients treated with gemcitabine (Sugiyama *et al*, 2007). In that study, 256 Japanese patients who had not previously received gemcitabine were enrolled. In our study, we defined the haplotype without amino-acid changes as the *1 group, and haplotypes harbouring the 79A>C and 208G>A were designated *2 and *3, respectively. The relationships between the diplotype groups and the pharmacokinetic parameters of gemcitabine are summarised in Table 2. The data clearly showed a haplotype *3-dependent decrease in gemcitabine clearance (CL m^−2^) and increases in peak concentration (*C*_max_) and area under the curve (AUC) values, although these parameters were not significantly influenced by haplotype *2. The values of AUC and CL m^−2^ observed in the patient with 208AA (*3/*3) were five-fold and one-fifth of the median of the 208GG (non*3/non*3) group, respectively (Figure 2). Then, associations of haplotype *3 with toxicities were analysed. Nadir grades of neutrophil counts were compared between the patient groups with or without haplotype *3 under individual therapeutic regimens. Although there were no significant differences in the incidences of grade ⩾3 neutropaenia between the two groups receiving gemcitabine monotherapy, grade ⩾3 neutropaenia occurred more frequently in the group with haplotype *3 than in the group without haplotype*3 when gemcitabine was administered with carboplatin, cisplatin, or 5-fluorouracil. We concluded that haplotype *3 harbouring 208G>A decreased the clearance of gemcitabine, and increased the incidence of neutropaenia when patients were coadministered platinum-containing drugs or 5-fluorouracil. Indeed, the patient with *CDA* 208AA developed severe myelosuppression with severe gastrointestinal toxicities after gemcitabine plus cisplatin combination therapy (Yonemori *et al*, 2005). Extra caution may be necessary if patients carrying a *3 allele, especially those who are homozygous for *3, are treated with gemcitabine. On the other hand, Vasile *et al* (2006) recently examined the correlation between *CDA* SNPs and clinical efficacy in 61 NSCLC patients treated with gemcitabine alone or gemcitabine plus cisplatin, and reported that the patients with *CDA* 79AA (*n*=21) showed a significantly better response rate and progression-free survival than those with *CDA* 79AC or 79CC (*n*=40) (response rate: 52.4 *vs* 20%; median progression-free survival: 8.0 *vs* 2.5 months; *P*=0.0136). Further functional and clinical studies focusing on these *CDA* SNPs are required.

With regard to gene expression, Ganti *et al* (2006) investigated the gene expression of CDA in bone marrow mononuclear cells in 71 patients with advanced solid tumours, and reported that patients with a lower relative gene expression of CDA tended to show a higher incidence of grades 2–4 haematological toxicity during gemcitabine therapy. Recently, some additional interesting results have been reported by Bengala *et al* (2005), who performed a phase I study of gemcitabine infusion at a fixed dose rate in patients with pancreatic cancer, and also investigated the relationship between CDA mRNA expression in peripheral blood mononuclear cells and clinical outcome. They reported that patients with a lower gene expression level of CDA showed significant longer overall survival than those with a higher expression level (median, 8.5 *vs* 3.7 months; *P*=0.03). On the other hand, as to expression in tumour tissues, Giovannetti *et al* (2006) reported that multivariate analysis failed to show any prognostic significance of CDA mRNA expression in 81 patients with pancreatic cancer receiving gemcitabine.

## DEOXYCYTIDINE KINASE

Deoxycytidine kinase is the rate-limiting enzyme for the intracellular phosphorylation of gemcitabine to its active phosphate form. Therefore, DCK may play an important role in sensitivity to gemcitabine. A clear correlation between DCK activity and gemcitabine sensitivity in tumour xenografts has been reported (Kroep *et al*, 2002).

Haplotype analysis in the 5′ regulatory region (−360C>G and −201C>T) suggested that −360C/−201C and −360G/−201T had almost complete linkage disequilibrium, and a functional study revealed that patients carrying the −360CG/−201CT and −360GG/−201TT genotypes expressed significantly higher levels of DCK mRNA than patients carrying the −360CC/−201CC genotype (Shi *et al*, 2004). Then the relationship between *DCK* SNP haplotypes and event-free survival in 122 patients with acute myeloid leukaemia treated with cytarabine was analysed, and slight but statistically significant prolongation of event-free survival time in the group with −360CG/−201CT and −360GG/−201TT over the group with −360CC/−201CC (2-year event-free survival rate, 30.7 *vs* 23.2%; *P*=0.0423) was observed. Recently, Joerger *et al* (2006) detected two nonsynonymous SNPs in a Caucasian population, 364C>T (Pro122Ser) and 727A>C (Lys243Gln), but their clinical relevance has not yet been clarified.

Recent clinical studies have also shown an association between tumoral DCK expression level and clinical outcome. Sebastiani *et al* (2006) investigated the relationship between the clinical outcome of pancreatic cancer patients treated with gemcitabine-based chemotherapy and immunohistochemical expression of DCK in cancer tissues. They reported that patients whose tumours showed low DCK expression (*n*=9) had significantly shorter overall survival than those whose tumours showed high expression (*n*=23) (median, 14.6 *vs* 21.7 months; *P*<0.009). They also sequenced the entire DCK-encoding gene in 17 human pancreatic cancer cell lines and nine samples of cancer tissue from patients, but no mutations were identified. Mey *et al* (2006) administered gemcitabine intravesically to 12 patients with bladder cancer, and reported that the mean expression of mRNA in the tumours was significantly higher in patients who achieved a complete pathological response than in those who did not. On the other hand, Seve *et al* (2005) reported that immunohistochemical expression of DCK protein in tumours was not significantly correlated with the survival of NSCLC patients treated with gemcitabine-based chemotherapy.

## 5′-NUCLEOTIDASE

Since phosphorylated metabolites of gemcitabine are reduced by cellular 5′-NT, the activity level of 5′-NT may be one of the factors affecting the clinical outcome of gemcitabine therapy. Using malignant cells obtained from 43 NSCLC patients receiving gemcitabine-based chemotherapy, Seve *et al* (2005) applied immunohistochemical methods to assess the abundance of proteins involved in gemcitabine pathways, including cN-II, one of the cytosolic nucleotidases that have been shown to be predictive factors in patients with acute myeloid leukaemia (AML) receiving cytarabine. They reported that cN-II was expressed in 86% of the patients, and that among various proteins investigated, only the level of cN-II was significantly correlated with overall survival (*P*=0.02). Since low levels of cN-II were associated with a poor prognosis in NSCLC patients receiving gemcitabine and with a better prognosis in AML patients receiving sytrabine (Seve *et al*, 2005), further studies are necessary to confirm the usefulness of cN-II as a prognosis factor.

## RIBONUCLEOTIDE REDUCTASE

Ribonucleotide reductase is the rate-limiting enzyme of the DNA synthesis pathway and converts ribonucleoside diphosphate to deoxyribonucleoside diphosphate, which is essential for DNA synthesis and repair. Ribonucleotide reductase consists of two subunits, ribonucleotide reductase M1 (RRM1) and ribonucleotide reductase M2 (RRM2).

Kwon *et al* (2006) investigated the association between polymorphisms of *RRM1* and gemcitabine chemosensitivity *in vitro* using 62 human cancer cell lines. When the association between these SNPs and gemcitabine IC_50_ was examined, only cell lines with *RRM1* 2232G>A showed a tendency to be more chemosensitive to gemcitabine, although none of the differences reached a statistically significant level. Bepler *et al* (2005) analysed the *RRM1* promoter for polymorphism, and discovered two SNPs, *RRM1* −37C>A and −524T>C. There was a strong linkage between these SNPs, and −37CC in combination with −524TT was the most frequently observed allelotype, accounting for 42.4% of the ethnically diverse population of 1129. They investigated *RRM1* promoter allelotypes and the outcomes of patients who had undergone surgical resection for NSCLC. It was found that patients with the −37CC/−524TT allelotype had better overall and disease-free survival than patients with the −37AC/−524CT allelotype (median overall survival, 80 *vs* 46 months; *P*=0.06, median disease-free survival, 74 *vs* 36 months; *P*=0.03). However, no association between allelotype and tumoral RRM1 expression was found.

Rosell *et al* (2004b) examined the potential correlation of RRM1 mRNA expression in specimens of NSCLC resected from 67 patients who had been treated with neoadjuvant gemcitabine/platinum. They found a good correlation between RRM1 expression in tumours and survival: significant differences in median survival were observed between the 17 patients in the bottom quartile of RRM1 expression and the 15 in the top quartile (median, 52 *vs* 26 months; *P*=0.018). They also reported similar results in patients with advanced NSCLC treated with gemcitabine/cisplatin therapy (Rosell *et al*, 2004a). Patients with low RRM1 mRNA expression levels had significantly longer median survival than those with high levels (median, 13.7 *vs* 3.6 months; *P*=0.009). Bepler *et al* (2006) also reported that increased RRM1 expression resulted in resistance to gemcitabine both *in vitro* and clinically. They found that the gemcitabine IC_50_ of lung cancer cell lines with increased RRM1 expression was higher than that of cell lines with decreased RRM1 expression, and the results they obtained in a prospective phase II clinical trial in patients with advanced NSCLC showed a significant inverse correlation between RRM1 expression and disease response to gemcitabine and carboplatin therapy (*P*=0.002 and *r*=−0.498). Therefore, tumoral RRM1 expression may be a useful marker of outcome in NSCLC patients receiving gemcitabine-based chemotherapy.

With regard to RRM2, the association between its genetic polymorphisms and resistance to gemcitabine has not been reported. Duxbury *et al* (2005) demonstrated an association of RRM2 overexpression with gemcitabine chemoresistance in pancreatic adenocarcinoma cells: the gemcitabine IC_50_ was four times higher in RRM2 recombinant than with an empty vector (*P*<0.05). Goan *et al* (1999) selected a gemcitabine-resistant cell line KB-GEM (IC_50_=32 *μ*M) from human oropharyngeal epidermoid carcinoma KB cells (IC_50_=0.3 *μ*M), and found that RRM2 mRNA (nine-fold) and protein (two-fold) were overexpressed in KB-GEM in comparison with the parental KB cells.

## DEOXYCYTIDYLATE DEAMINASE AND UMP/CMP KINASE

Gemcitabine monophosphate is inactivated to dFdUMP by DCTD. A few SNPs including a nonsynonymous one, *DCTD* 172A>G (Asn58Asp), have been reported (Table 1; Fukunaga *et al*, 2004; Gilbert *et al*, 2006). Recombinant Asp58 DCTD was reported to have 11% of wild-type activity for dFdCMP. dFdCMP is further phosphorylated to dFdCDP by UMP/CMP kinase, which is ubiquitously present in human tissues (van Rompay *et al*, 1999). To date, neither association of genetic polymorphisms nor expression of either DCTD or UMP/CMP kinase with clinical outcome of gemcitabine treatment has been demonstrated.

## DNA REPAIR

As the main mechanism of action of gemcitabine is potent inhibition of DNA synthesis, DNA repair may play an important role in gemcitabine-mediated cell death. Recently, Li *et al* (2006) investigated 13 SNPs of eight DNA damage response and repair genes in 92 patients with resectable pancreatic cancer treated with neoadjuvant gemcitabine-based chemotherapy. They found that *RecQ1* 1596(^*^6), *Rad54L* 2190C>T, and *ATM* IVS20-77 T>C genotypes had a significant effect on overall survival. The strongest genetic effect on survival was observed for *RecQ1* 1596(^*^6), with median overall survival times of 18.9 and 13.1 months for the AC and CC genotypes, respectively, compared with a mean survival time of 46.9 months for the AA wild type (*P*=0.001). De las Penas *et al* (2006) investigated the association of survival with genetic polymorphisms of various DNA repair genes in 135 cisplatin/gemcitabine-treated NSCLC patients at stage IIIB and IV. After adjusting for performance status, a significantly low hazard ratio (0.44) for carriers of *XRCC3* 722TT (241Met/Met) compared to carriers of 722CT (241Thr/Met) was demonstrated (*P*=0.01). With regard to the expression levels of DNA repair genes, Lord *et al* (2002) investigated the relationship between excision repair cross-complementing group 1 ERCC1 expression in tumours with response or overall survival in NSCLC patients treated with cisplatin/gemcitabine. They failed to show any significant association between ERCC1 expression and response, but reported that low expression of ERCC1 in tumours was associated with longer survival (61.6 *vs* 20.4 weeks in the low and high expression groups, respectively). Bepler *et al* (2006) also found a similar trend for the relationship between ERCC1 expression and NSCLC response. Cytotoxic synergism has been demonstrated between gemcitabine and cisplatin through downregulation of ERCC1 activity by gemcitabine (Lord *et al*, 2002).

## CONCLUSION AND FUTURE DIRECTIONS

In this article, we have reviewed recent genetic studies of gemcitabine. The impact of genetic polymorphisms as well as tumour-specific expression of mRNA/proteins on gemcitabine efficacy and toxicity has been described. Looking at these data, tumour-specific expression of ENT1, RRM1 or ERCC1, or some DNA repair genetic polymorphisms appear to be promising indicators of prognosis in patients receiving gemcitabine chemotherapy, although prospective pharmacogenetic-based clinical studies will be necessary to clarify the usefulness of these biomarkers in patients receiving gemcitabine-based chemotherapy. With regard to adverse reactions caused by gemcitabine, the expression level or genetic polymorphism of *CDA* seems to be a good predictor. SNP, *CDA* 208A>G, or CDA expression level may be candidate biomarkers for individualised gemcitabine-based chemotherapy to avoid severe toxicity, at least in Japanese and some African populations in which considerable numbers of homozygote carriers exist, as is the case for UGT1A1*28 for irinotecan and *TPMT* genotypes for thiopurine drugs.

## Figures and Tables

**Table 1:**
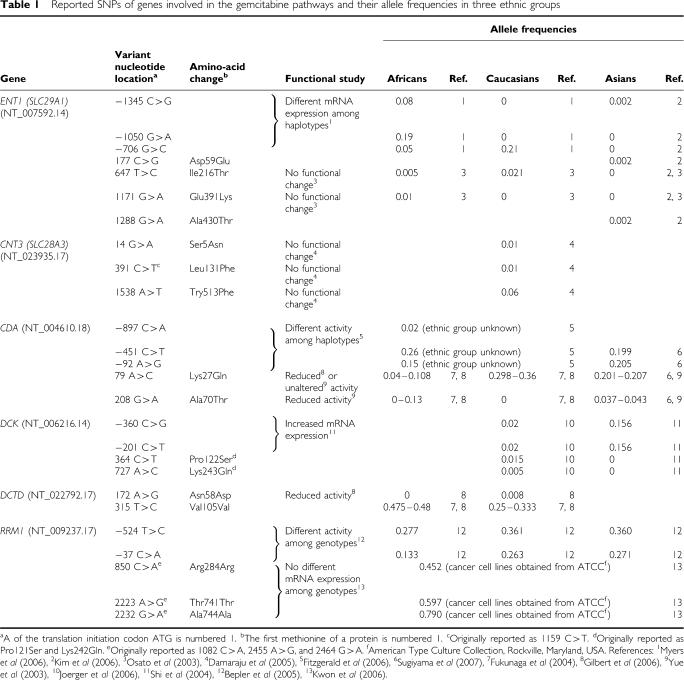
Reported SNPs of genes involved in the gemcitabine pathways and their allele frequencies in three ethnic groups

**Table 2:** Pharmacokinetic parameters of gemcitabine in the patient groups categorized according to diplotypes

	**Mean±s.d.**			
**Diplotype**	*1/*1 (*n*=148)	*2/*1 (*n*=69)	*2/*2 (*n*=15)	
				
**PK parameter**				***P*-value^a^**
*C*_max_ (mg ml^−1^)	22.7±6.3	22.9±6.4	24.1±5.5	0.52
AUC (h *μ*g ml^−1^)	10.1±2.5	9.8±2.3	9.8±1.5	0.46
CL m^−2^ (l h^−1^ m^−2^)	105.8±31.1	107.2±27.2	103.3±19.2	0.99
				

	**Mean±s.d.**			
**Diplotype**	*1/*1 (*n*=148)	*3/*1 (*n*=13)	*3/*3 (*n*=1)	
				
**PK parameter**				***P*-value^a^**
				
*C*_max_ (mg ml^−1^)	22.7±6.3	26.8±5.9	46.4	5.94E-04
AUC (h *μ*g ml^−1^)	10.1±2.5	12.7±2.6	52.9	6.66E-13
CL m^−2^ (l h^−1^ m^−2^)	105.8±31.1	82.6±24.9	18.9	7.77E-04

a *P*-value of a correlation test. Multiplicity is adjusted by the false-discovery rate.
